# Preserved neurovascular coupling in early multiple sclerosis is associated with reduced white matter lesion burden

**DOI:** 10.1177/17562864261481753

**Published:** 2026-08-24

**Authors:** Cristina Duque, João Sargento-Freitas, Roumen Balabanov, Bruce Cohen, Farzaneh Sorond

**Affiliations:** 1 Faculty of Medicine, 58411 Coimbra University, Coimbra, Portugal; 2 Neurology Department, Hospital Pedro Hispano, 379989 Unidade Local de Saúde de Matosinhos, Matosinhos, Portugal; 3 Neurology Department, Unidade Local de Saúde de Coimbra, 58411 Hospitais da Universidade de Coimbra, Coimbra, Portugal; 4 Neurology Department, Feinberg School of Medicine, 3270 Northwestern University, Chicago, USA

**Keywords:** multiple sclerosis, neurovascular coupling, transcranial doppler ultrasound, MRI

## Abstract

**Background:**

Multiple sclerosis (MS) is a chronic autoimmune disorder characterized by inflammation, demyelination and axonal degeneration of the central nervous system. Whether these pathological changes impact neurovascular coupling (NVC), which can be compromised in various neurodegenerative disorders, remains unclear in MS.

**Objectives:**

To investigate the relationship between NVC, clinical characteristics and neuroimaging measures in patients with MS.

**Design:**

We conducted a cross-sectional study of patients with MS recruited from the Comprehensive Multiple Sclerosis Clinic at Northwestern Medicine, Chicago, USA.

**Methods:**

Seventy-six patients with MS underwent NVC assessment using transcranial Doppler (TCD) ultrasound in the middle cerebral artery (MCA) and posterior cerebral artery (PCA) territories. Clinical assessment included manual dexterity, cognitive function and gait. Brain magnetic resonance imaging (MRI) quantified white matter hyperintensity (WMH), gray matter (GM) and white matter (WM) volumes. Associations were examined using multivariate linear regression, adjusting for demographic and vascular risk factors, and stratified by disability and cognitive performance.

**Results:**

Higher NVC in the PCA territory correlated with lower WMH burden (r=−0.32, p=0.02), particularly in patients with lower disability (Expanded Disability Status Scale <2). This association remained significant after adjustment (β=−0.071, 95% CI [−0.135, −0.007], p=0.031). Higher NVC PCA was also associated with lower WMH in patients with better executive (β=−0.104, 95% CI [−0.176, −0.033], p=0.007) and global cognitive scores (β=−0.116, 95% CI [−0.222, −0.009], p=0.035). No association was found with GM volume.

**Conclusion:**

Preserved PCA neurovascular function is associated with lower WM lesion burden, measured using conventional MRI, in MS patients with lower disability and better cognition, a finding that may inform risk stratification and guide future therapeutic targets.

## Plain language summary

Multiple sclerosis (MS) is a disease where the immune system damages the protective covering of nerve fibres in the brain. This damage shows up as bright spots on brain scans. Treatments can reduce immune attacks, but we do not fully understand why some patients develop more brain damage than others. The brain normally increases blood flow to areas that are working hard, a process that ensures brain cells get enough oxygen. This process can break down in brain diseases, but it has not been well studied in MS. We studied 76 people with MS. Using ultrasound on the skull, we measured how well blood flow increased in brain arteries when participants looked at a flickering screen or performed memory tasks. We then compared these measurements with brain scan results showing areas of damage, and with each patient’s level of disability and thinking ability. In patients with milder MS and better thinking abilities, those whose brains were better at controlling blood flow had fewer damaged areas on their scans. This link remained after accounting for age, sex, and common health conditions such as high blood pressure. In patients with greater disability, this relationship was not seen. Measuring how well the brain adjusts its blood flow could help identify MS patients who are more resilient to brain damage at earlier disease stages. This simple, low-cost ultrasound test could complement brain scans and help doctors identify patients who might benefit from closer monitoring or different treatment approaches. Further studies following patients over time are needed to confirm these findings.

## 1. Introduction

Multiple sclerosis (MS) is a chronic autoimmune disorder characterized by inflammation and demyelination of the central nervous system (CNS), resulting in widespread neurodegeneration.^1^ Disability accumulation in MS can occur at any stage, driven by both relapse-associated worsening and progression independent of relapse activity.^2^ Neurodegeneration is thought to be the predominant mechanism in progressive forms of MS,^3^ where anti-inflammatory and immunosuppressive therapies have limited efficacy.^4^

MS pathogenesis remains incompletely understood, and while T-cell mediated responses are crucial in focal lesion formation,^5^ mechanisms of axonal degeneration and progressive MS are less clear.^6^ Vascular abnormalities in MS include increased risk of cerebral small vessel disease^7^ (CSVD) and reduced cerebral perfusion.^8^ Converging inflammatory pathways leading to oxidative stress and endothelial dysfunction play a role in cerebrovascular injury.^9^ Moreover, focal perivascular inflammation^10^ and reduced axonal activity^11^ are thought to impair brain perfusion and amplify vascular damage in MS.^12^

Neurovascular coupling (NVC) is defined by a regional increase in cerebral blood flow (CBF) in response to neuronal activation and mediated by dynamic changes in blood vessels.^13^ The neurovascular unit (NVU) is comprised of vessels, neurons and glia. NVC dysfunction is a significant feature of neurodegenerative disorders.^14^ In MS, vascular damage has been described in both normal-appearing white matter (WM) and acute demyelinating lesions,^15,16^ suggesting a critical vascular role across the disease continuum.^17^ Key NVU components, including endothelial cells, astrocytes, pericytes and microglia are known to be altered in MS.^18^ Whether neurovascular dysregulation is a primary contributor to MS pathophysiology or a consequence of the pathophysiological process, remains unknown.

Transcranial Doppler ultrasound (TCD) is a robust, non-invasive technique to measure CBF velocity^19^ for assessment of cerebral vascular responses to physiological challenges, including motor or cognitive activation (NVC), that are known to be regulated at the level of arterioles or resistance vessels.^20,21^ TCD-based NVC measurements provide a cost-effective approach with high-temporal resolution to assess neurovascular health. Investigating CBF and NVC as proxies for cerebrovascular hemodynamic efficiency may reveal mechanisms contributing to MS progression, where inflammation plays a lesser role in disability accumulation.^6^ Our study undertook a comprehensive investigation of NVC using TCD in MS, exploring associations with clinical measures (upper extremity function, cognition, and gait), as well as structural brain measures (magnetic resonance imaging; MRI) specific to CSVD.

## 2. Material and methods

### 2.1. Study design and participants

This cross-sectional study recruited participants from the Comprehensive Multiple Sclerosis Clinic at Northwestern Medicine. All participants fulfilled the 2017 McDonald diagnostic criteria for MS,^22^ with diagnoses reconfirmed according to the 2024 criteria,^23^ and had normal visual acuity. Participants were excluded if they had clinical relapses or steroids use within 3 months of screening, history of stroke or other major brain injuries (head trauma, brain tumor or degenerative neurological diseases), significant psychiatric disorders including major depression, unsuitable acoustic temporal bone window, extra or intracranial stenosis exceeding 50%, or inability to cooperate or provide informed consent. The study was approved by the Northwestern University Institutional Review Board. All participants provided written informed consent in accordance with the Declaration of Helsinki.

### 2.2. Data collection

Demographic details, current medications, time from MS onset, disease severity quantified by the Expanded Disability Status Scale^24^ (EDSS), and vascular risk factors (hypertension, diabetes mellitus, dyslipidemia, smoking status and body mass index) were extracted from each participant’s Electronic Health Records. This study was conducted under Northwestern University IRB protocol STU00201651-MOD0002, and the ethical approval was granted specifically for the current study. Approval was obtained on 2 February 2017, prior to the start of participant recruitment. The first participant was enrolled on 6 February 2017 and the data collection cut-off date was 30 June 2018; recruitment began only after approval was in place. The approval and the written informed consent obtained from each participant therefore covered the entire study period and all data used, including participants recruited through 2018. No data were collected or used outside the period authorized by this approval. All participants completed a single-visit comprehensive testing protocol including TCD assessment, manual dexterity evaluation, cognitive testing and gait assessment. MRI data were obtained from the most recent clinical imaging study conducted within one year of the study visit as part of routine clinical follow-up.

### 2.3. Neurovascular coupling

NVC was assessed independently for the middle cerebral artery (MCA) and the posterior cerebral artery (PCA) using TCD ultrasonography (Digi-LiteTM - Rimed Ltd). Mean cerebral blood flow velocity (BFV) was continuously recorded at rest and during task performance. Bilateral 2 MHz probes were secured over each temporal bone using a Mueller-Moll probe fixation device. The M1 segment of each MCA was insonated at 50 to 60 mm depth, while the P2 segment of each PCA at 60 to 70 mm.^19^ Simultaneous physiological monitoring included heart rate (HR) from electrocardiogram (ECG) and beat-to-beat arterial pressure (BP) monitoring (Finapres NOVA, Finapres Medical Systems BV), as previously described.^25^ The velocity waveform envelope was derived from fast-Fourier analysis of the Doppler frequency signal, digitized at 500 Hz, and displayed simultaneously with BP and ECG signals. Participants refrained from caffeine and heavy meals for at least 2 hours prior to data collection. All measurements were obtained in a dimly lit room with controlled ambient temperature, with participants seated upright.

NVC in the MCA was evaluated by measuring changes in BFV during N-Back test performance (1-Back and 2-Back) relative to a control task (Identify letter X), as reported before.^26^ For each 120-second task block, mean BFV values were extracted from the middle 100-second time window to allow for BFV stabilization. Mean percentage change for each MCA was calculated using: (BFVNB-BFVIDX)/(BFVIDX) x 100%; where BFVNB corresponds to BFV during N-back task, and BFVIDX to BFV during Identify the Letter X control period. The 2-Back task was selected as primary outcome measure due to greater CBF increase, yielding more sensitive NVC measurement.^25–27^

For the PCA territory, NVC was assessed using a visual stimulation paradigm of 10 cycles, each comprising 20 seconds of eyes-closed rest followed by 40 seconds of stimulation with a 10 Hz flickering checkerboard.^28^ NVC PCA response was quantified as the maximum mean BFV change, calculated using: (maximum BFV – baseline BFV)/baseline BFV x 100%.^28^ Baseline BFV was defined as mean of a 5-second stable measurement preceding stimulation.^29^ As no significant interhemispheric differences were observed, right and left values were averaged for each artery.

### 2.4. Manual dexterity, cognitive function and gait

Manual dexterity was measured using the 9-Hole Peg Test (9-HPT), a quantitative test of upper extremity function in MS.^30^ Participants were instructed to individually transfer pegs from a container to nine holes on a board; once filled, pegs were removed as quickly as possible and returned to the container.^31^ The procedure was executed twice on each arm and the score, in seconds, was averaged across sides.

Cognitive function was evaluated using a comprehensive neuropsychological battery, encompassing Montreal Cognitive Assessment Test (MoCA), Hopkins Verbal Learning Test-Revised (HVLT-R), Trails Making Tests A (TMT-A) and B (TMT-B), Digit Span Forward (DSF) and Backward (DSB), and Paced Auditory Addition Test (PASAT-3). Four cognitive composite domains were created using standardized z-scores: attention, executive function, memory and global cognition.^32^ TMT-A and TMT-B z-scores were inverted to ensure directional consistency. Attention composite incorporated TMT-A and DSF scores, while executive function combined TMT-B and DSB performance. Memory composite was derived from HVLT-R total and delayed recall scores. Global cognition composite integrated all previous tests along with MoCA and PASAT-3.

Gait analysis was conducted using a Zeno electronic walkway system (Protokinetics Zeno™ Walkway, 20 x 2-foot mat). Participants completed four walking trials: two single- and two dual-tasks, each with two passes across the mat in a continuous walking session over a 40-foot course. Turns were executed off the mat and excluded from analysis. For single-task walking, participants walked at normal pace then another trial was performed at a fast pace. For dual-task walking, participants walked at normal pace while verbally subtracting by 5, 3 or 1 from 500, as determined previously.^33^ A second dual-task trial was performed at a fast pace during serial subtraction. Step velocity (cm/s) was measured automatically using the Protokinetics Movement Analysis Software.

### 2.5. Magnetic resonance imaging

MRI data were acquired from the most recent clinical MRI within one year of participant enrollment, with data excluded if there was evidence of clinical relapses within the preceding 3 months. MRI protocol was performed on a Siemens Sola 1.5T scanner and included T1-weighted fat-saturated axial images (TR=760 ms, TE=10 ms, matrix=230 x 256, FOV=230 x 230 mm^2^, pixel size=1.0 x 0.9) and T2 fluid-attenuated inversion recovery sagittal images (TR=7000 ms, TE=424 ms, TI=2050 ms, matrix=256 x 256, FOV=250 x 250 mm^2^, pixel size=0.98 x 0.98). Global and regional brain volume segmentation were performed on T1 sequences using FreeSurfer neuroimaging analysis suite (v7.4.0., https://surfer.nmr.mgh.harvard.edu). White matter hyperintensities (WMH) volumes were derived from the T2 sagittal FLAIR sequences. Images were preprocessed using an adaptive non-local means filter for denoising^34^ and the N4 algorithm for intensity bias correction.^35^ The Lesion Prediction Algorithm^36^ from the Lesion Segmentation Toolbox (LST 2.0.15) for statistical parametric mapping was applied to cleaned images. Lesion masks were generated by thresholding voxel-wise lesion probability maps at >0% and manually corrected using Freeview in FreeSurfer. Analysis of Functional NeuroImages software was employed to generate volumetric data, including intracranial volume (ICV), gray matter (GM) and WM volumes. To account for variations in individual head sizes, all volumes were normalized by respective ICV. This procedure was executed for each participant, with all masks undergoing a secondary review for quality assurance.

### 2.6. Statistical analysis

Normality of variables was evaluated using Shapiro-Wilk test and histograms visual inspection. Spearman’s correlation coefficient assessed relationships between NVC values and manual dexterity, cognition, gait and MRI metrics. Significant correlations between NVC and functional or structural outcomes were examined using multivariate linear regression. MRI-related analyses were adjusted for age, sex, race, EDSS, MS duration and vascular risk factors (VRF). VRF were quantified on a 5-point score,^32^ allocating one point each for hypertension, diabetes, current or past smoking, total cholesterol ≥200 mg/dL and body mass index (BMI) ≥25 kg/m^2^. To investigate NVC disability-dependent associations, the sample was stratified by the median EDSS score (2) and tested for interaction (EDSS x NVC). To examine whether cognitive performance influenced the association between NVC and brain structure, sample stratification was done based on cognitive percentile scores across each composite domain. Statistical significance was set at a p-value <0.05 (two-sided). Analyses were conducted using SPSS v. 28, while graphics were generated using Python v. 3.11.6.

## 3. Results

Seventy-six participants completed data collection. Baseline characteristics are in Table 1. Seventy-one participants (93%) had relapsing-remitting (RR) MS, with median disease duration of 8 years (IQR ±11). The majority received infusion therapy (59%), mostly natalizumab (33%). At least one VRF was present in 50 (66%) participants. High BMI (≥25 kg/m^2^) was the most common VRF (57%). All participants with hypertension (11%), diabetes (11%) and dyslipidemia (12%) were on treatment.

**Table 1.** Participants baseline characteristics: Overall sample and across disability groups.

​	All	EDSS category		p
	n=76	Low n=33	High n=43	
**Demographics**				
Age, years^+^	44 (10)	39 (10)	49 (9)	<0.001*
Male, n (%)	21 (28)	7 (21)	14 (33)	0.273
White, n (%)	71 (93)	33 (100)	38 (88)	0.043*
**Multiple Sclerosis Clinical Characteristics**				
RR, n (%)	71 (93)	33 (100)	38 (88)	0.128
Disease duration^++^	8 (11)	5 (10)	10 (14)	0.042*
EDSS^++^	2.0 (4.0)	1.0 (2.0)	4.0 (4.0)	<0.001*
Infusion therapy, n (%)	45 (59)	19 (57)	26 (60)	0.799
*Natalizumab*	25 (33)	13 (39)	12 (28)	0.291
*Ocrelizumab*	19 (25)	6 (18)	13 (30)	0.229
*Alemtuzumab*	1 (1)	0 (0)	1 (2)	0.378
Other treatments, n (%)	24 (32)	11 (33)	13 (31)	0.773
*Dimethyl fumarate*	6 (8)	3 (9)	3 (7)	0.735
*Mycophenolate mofetil*	5 (7)	2 (6)	3 (7)	0.947
*Glatiramer acetate*	4 (5)	2 (6)	2 (5)	0.785
*Interferon-β*	4 (5)	1 (3)	3 (7)	0.445
*Teriflunomide*	3 (4)	3 (9)	0 (0)	0.044*
*Fingolimod*	2 (3)	0 (0)	2 (5)	0.209
No treatment, n (%)	7 (9)	3 (9)	4 (9)	0.975
**Vascular Risk Factors**				
History of hypertension, n (%)	8 (11)	1(3)	7 (16)	0.062
History of diabetes, n (%)	8 (11)	3 (9)	5 (11)	0.721
Current Smoker, n (%)	6 (8)	3 (9)	3 (7)	0.735
Dyslipidemia, n (%)	9 (12)	2 (6)	7 (16)	0.172
BMI ≥25 kg/m^2^, n (%)	43 (57)	14 (42)	29 (67)	0.029*
**Neurovascular Coupling**				
MCA BFV, cm/s, % change^++^	3.34 (6.06)	5.04 (8.08)	2.06 (4.50)	0.117
PCA BFV, cm/s, % change^++^	5.37 (10.86)	5.42 (10.54)	5.09 (11.5)	0.991
**Manual Dexterity**				
9HPT, dominant hand, s^++^	21 (7)	19 (3)	24 (9)	<0.001*
9HPT, non-dominant hand, s^++^	22 (8)	21 (3)	24 (12)	<0.001*
**Cognitive Parameters**				
Education, years^++^	16 (4)	16 (2)	16 (4)	0.241
Attention score^+^	0.01 (0.83)	0.35 (0.68)	-0.25 (0.85)	0.001*
Executive score^+^	0.01 (0.86)	0.29 (0.73)	-0.21 (0.90)	0.011*
Memory score^+^	-0.03 (0.96)	0.27 (0.25)	-0.26 (1.03)	0.016*
Global score^+^	0.01 (0.65)	0.24 (0.53)	-0.17 (0.69)	0.005*
**Gait Velocity**				
Self-pace, cm/s^++^	121 (47)	136 (27)	95 (44)	<0.001*
Fast-pace, cm/s^++^	161 (56)	177 (42)	139 (59)	<0.001*
Self-pace dual-task, cm/s^++^	79 (53)	86 (40)	61 (51)	<0.001*
Fast -pace dual-task, cm/s^++^	143 (59)	151 (33)	108 (59)	<0.001*
**MRI Measures**				
White matter volume^+^	0.30 (0.02)	0.30 (0.03)	0.29 (0.02)	0.183
WM hyperintensities^++^	0.003 (0.005)	0.002 (0.005)	0.004 (0.009)	0.050
Gray matter volume^+^	0.41 (0.03)	0.43 (0.04)	0.40 (0.02)	0.006*
Cortical GM volume^+^	0.31 (0.02)	0.32 (0.03)	0.30 (0.02)	0.003*
Subcortical GM volume^+^	0.04 (0.01)	0.04 (0.01)	0.04 (0.00)	0.036*
Time TCD to MRI, days^++^	52 (135)	71 (152)	47 (118)	0.527

P-values were calculated using t test or Mann-Whitney U test for continuous variables, and χ^2^ test for categorical variables. EDSS was divided at the median of the sample: low < 2 and high ≥ 2. Dyslipidemia defined by a total cholesterol ≥ 200 mg/dL. MRI measures normalized for intracranial volume.

N=5 missing for NVC MCA and N=3 missing for NVC PCA. N=11 missing for GM and WM measures, N=21 missing for WM hyperintensities. N=1 missing for executive cognitive domain. N=7 missing for gait velocity self-pace, N=13 for fast pace, N=9 missing for gait dual task and N=16 for fast dual task. N=3 missing for 9HPT dominant hand and N=3 missing for 9HPT non-dominant hand.

Abbreviations: BMI= Body Mass Index; EDSS= Expanded Disability Status Scale; GM= gray matter; MCA= middle cerebral artery; BFV= mean blood flow velocity; MRI= magnetic resonance imaging; NVC= neurovascular coupling; PCA= posterior cerebral artery; RR= relapsing-remitting; s= seconds; WM= white matter; 9HPT= Nine-Hole Peg test.

^+^Values are presented as mean (standard deviation).

^++^Values are presented as median (interquartile range).

^*^p-value <0.05.

**Figure 1.**
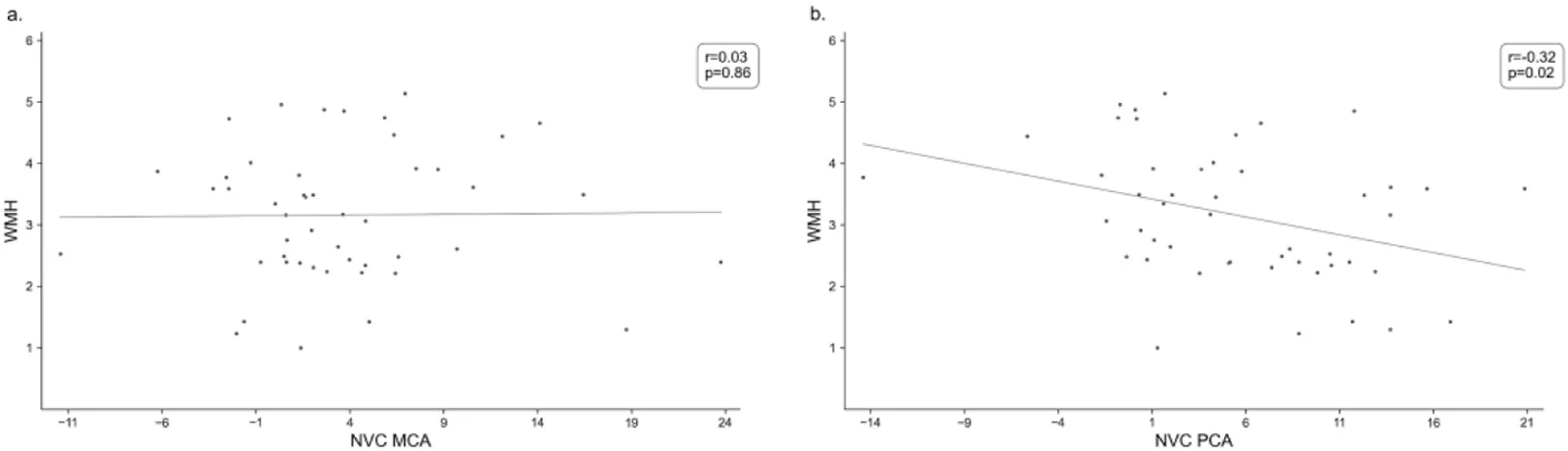
Relationship between neurovascular coupling and white matter hyperintensities. Scatterplots showing the relationship between NVC in the MCA and WMH (a) and NVC in the PCA and WMH (b). The regression line, correlation coefficient (r) with associated p-value are displayed. NVC MCA and NVC PCA in cm/s, % change; WMH in % ICV, WMH values were log-transformed, and the minimum value was normalized to 1 for visualization. Abbreviations: ICV= intracranial volume; MCA= middle cerebral artery; NVC= neurovascular coupling; PCA= posterior cerebral artery.

**Figure 2.**
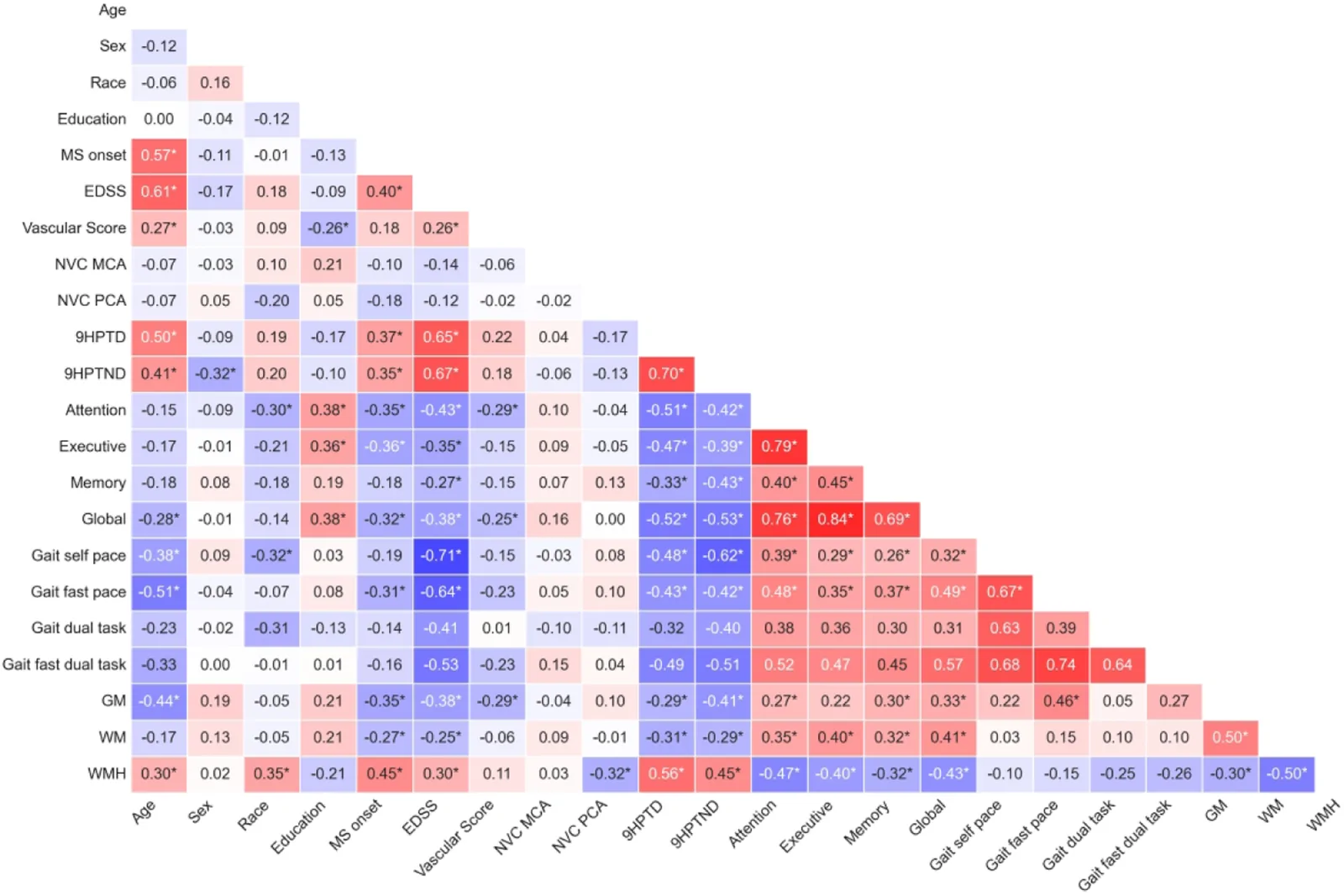
Correlation heatmaps of neurovascular coupling, clinical and neuroimaging variables. Heatmaps depicting the correlations between NVC and clinical measures, including manual dexterity, cognition, gait and MRI-based metrics. Age, gender, race, education, MS onset, EDSS and vascular score were also studied. Each square displays the Spearman’s correlation coefficient (r). Negative correlations are represented in blue, while positive correlations are shown in red. The intensity of the background color corresponds to the strength of the correlation, with darker shades of blue indicating stronger negative correlations and darker shades of red indicating stronger positive correlations. Age, education and MS onset in years; NVC MCA and NVC PCA in cm/s, % change; 9HPTD and 9HPTND in seconds; gait measurements in cm/s; GM, WM and WMH in % ICV. See Methods for detailed calculation procedures. *Indicates a P-value <0.05. Abbreviations: EDSS= Expanded Disability Status Scale; GM = gray matter; ICV= intracranial volume; MCA= middle cerebral artery; MRI= magnetic resonance imaging; MS= multiple sclerosis; NVC= neurovascular coupling; PCA= posterior cerebral artery; WM = white matter; WMH = white matter hyperintensities; 9HPTD= Nine-Hole Peg test, dominant hand; 9HPTND= Nine-Hole Peg test, non-dominant hand.

Figure 1(a) and (b) present scatterplots and regression lines illustrating the relationship between NVC in MCA and PCA territories and WMH burden. PCA NVC showed significant inverse correlation with WMH burden (r=-0.32, p=0.02), whereas there was no significant correlation for MCA NVC (r=0.03, p=0.86). After adjusting for demographic factors (age, sex and race), clinical MS characteristics (disease duration and EDSS) and VRF, the association between NVC PCA and WMH was no longer significant (β= -0.021, 95% CI [-0.055,0.012], p = 0.206). No significant correlations were observed between other functional (manual dexterity, cognitive performance and gait speed) or MRI metrics and NVC in the MCA or PCA territories (Figure 2).

To explore whether the relationship between NVC and WMH varied across disability levels, we conducted a stratified analysis by dividing the sample according to the median EDSS score of 2 (Table 1). NVC responses did not differ significantly between lower (EDSS <2) and higher disability groups (EDSS ≥2) (MCA 5 IQR ±8 vs 2 IQR ±5%; PCA 5 IQR ±11 vs 5 IQR ±12%, p=0.991). However, these groups had significant differences in clinical and imaging variables. The lower disability group had higher cognitive scores in every domain, better manual dexterity, faster gait speed for all tasks and greater GM volume (all p<0.05), while WM and WMH volumes showed no significant differences.

**Figure 3.**
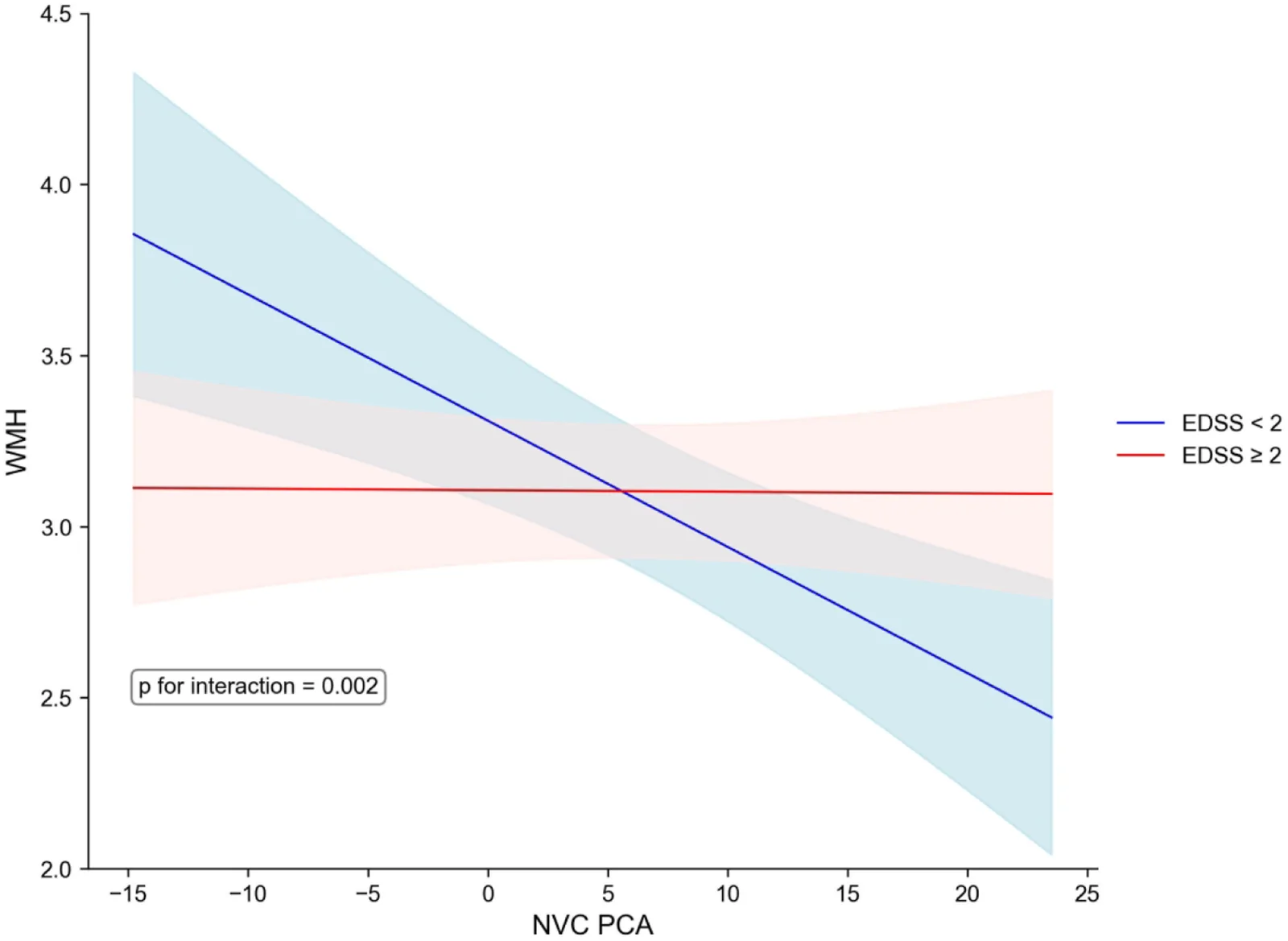
Interaction between neurovascular coupling and white matter hyperintensities stratified by disability status. Interaction plots showing the relationship between NVC and WMH stratified by disability group based on the median EDSS (2). The blue line represents MS individuals with lower disability (EDSS< 2) and the red line represents MS individuals with higher disability (EDSS≥ 2). Shaded areas indicate the 95% confidence intervals. A significant interaction effect is observed between NVC and EDSS status on WMH, with p=0.002. All analyses were adjusted for age, sex, race, education, years of MS duration, EDSS and vascular risk score. NVC PCA in cm/s, % change; WMH in % ICV, WMH values were log-transformed, and the minimum value was normalized to 1 for visualization. Abbreviations: EDSS= Expanded Disability Status Scale; MS= multiple sclerosis; NVC= neurovascular coupling; PCA= posterior cerebral artery; WMH= white matter hyperintensities.

Notably, in the lower disability group, a significant adjusted negative association between NVC PCA and WMH persisted (β= -0.071, 95% CI [-0.135, -0.007], p=0.031). This association was not observed in the higher disability group (β= 0.001, 95% CI [-0.038,0.040], p=0.949). Figure 3 shows the significant interaction (p=0.002) between EDSS groups and NVC PCA in relation to WMH burden. In the final adjusted model, the combination of NVC in the PCA territory, EDSS group, and vascular risk factors explained 49.8% of the variance in WMH burden and the overall fit was statistically significant (F (8,43) = 5.332, p<0.001).

Finally, to investigate whether the relationship between PCA NVC and WMH varied based on cognitive performance, we stratified the sample by cognitive percentile scores across each domain (attention, executive, memory, and global) (Table S1), adjusting for relevant covariates. In those with higher executive and global cognitive scores, higher NVC PCA was associated with lower WMH volume (β= -0.104, 95% CI [-0.176, -0.033], p=0.007 and β= -0.116, 95% CI [-0.222, -0.009], p=0.035, respectively) and, in those with higher global scores, with lower WM volume (β= -0.001, 95% CI [-0.002, -0.000], p=0.022) (Table 2). No association was found between NVC PCA and GM volume across any cognitive percentile.

**Table 2.** Neurovascular coupling PCA in relation to MRI measures, All participants and divided across low versus high cognition domains: Attention, executive, memory and global.

​	*β [95% CI]*					
	WM volume	*P* value	WM hyperintensities	*P* value	GM volume	*P* value
All	0.000 [-0.001, 0.000]	0.303	-0.022 [-0.064, 0.021]	0.305	0.000 [-0.001, 0.001]	0.976
Low Attention	0.000 [-0.001, 0.002]	0.509	-0.034 [-0.095, 0.028]	0.266	0.001 [-0.001, 0.002]	0.353
High Attention	-0.001 [-0.002, 0.000]	0.130	-0.043 [-0.136, 0.051]	0.350	-0.001 [-0.003, 0.001]	0.339
Low Executive	0.000 [-0.002, 0.001]	0.593	-0.004 [-0.062, 0.054]	0.891	0.000 [-0.001, 0.002]	0.780
High Executive	0.000 [-0.001, 0.001]	0.789	-0.104 [-0.176, -0.033]*	0.007	0.000 [-0.003, 0.002]	0.850
Low Memory	0.000 [-0.001, 0.002]	0.644	-0.028 [-0.092, 0.037]	0.376	0.000 [-0.001, 0.002]	0.866
High Memory	-0.001 [-0.002, 0.000]	0.072	0.009 [-0.079, 0.097]	0.834	0.000 [-0.002, 0.002]	0.807
Low Global	0.001 [-0.001, 0.002]	0.472	-0.015 [-0.087, 0.057]	0.663	0.000 [-0.002, 0.002]	0.515
High Global	-0.001 [-0.002, 0.000]*	0.022	-0.116 [-0.222, -0.009]*	0.035	-0.001 [-0.003, 0.001]	0.491

β represents unstandardized regression coefficients. All analyses were adjusted for age, sex, race, education, years of MS duration, EDSS and vascular risk score. NVC PCA in cm/s, % change; WMH in % ICV.

Abbreviations: CI= confidence interval; EDSS= Expanded Disability Status Scale; ICV= intracranial volume; GM= gray matter; MRI= magnetic resonance imaging; NVC= neurovascular coupling; PCA= posterior cerebral artery; WM= white matter.

^*^Indicates a P-value <0.05.

## 4. Discussion

**Figure 4.**
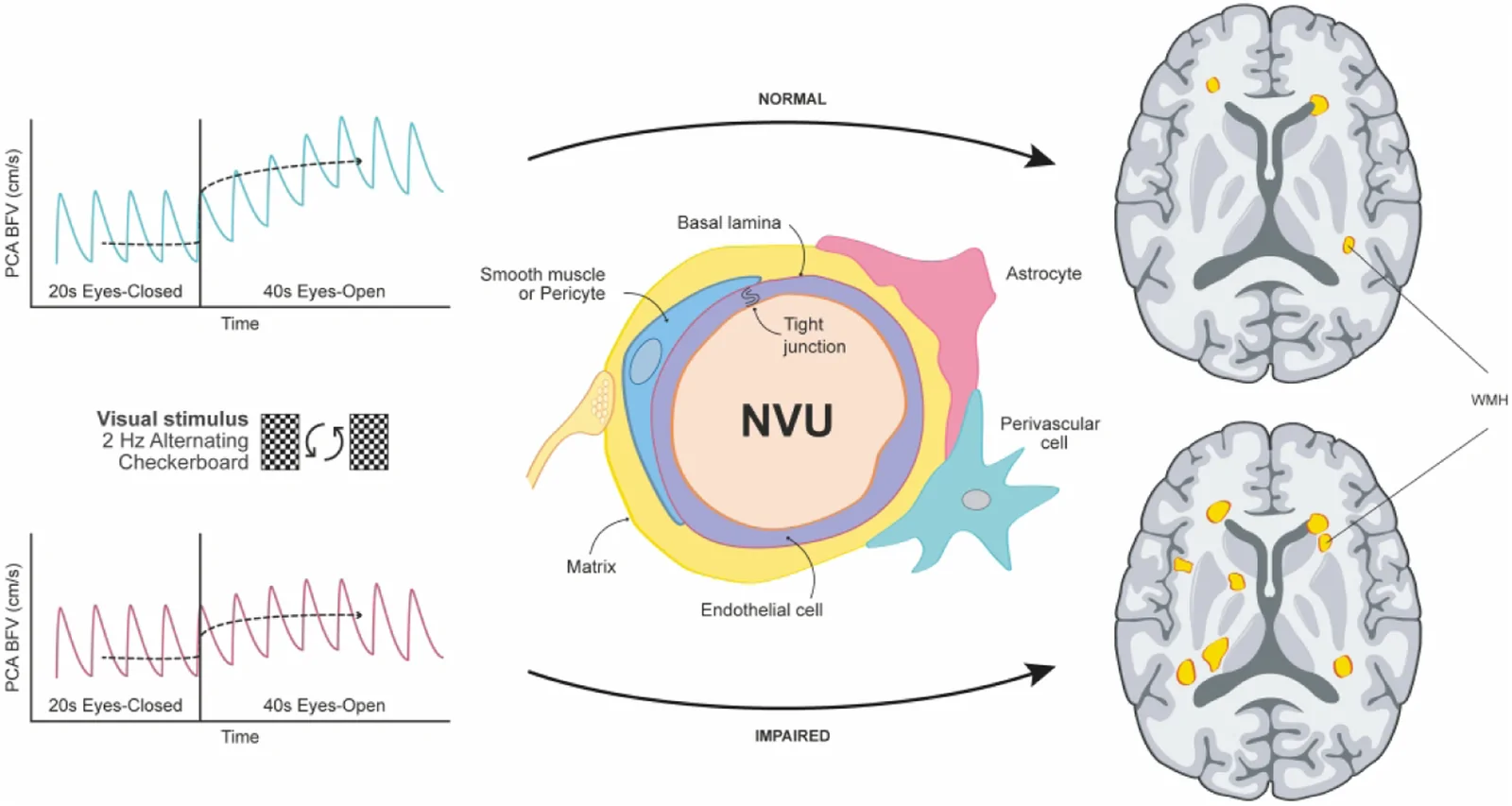
Schematic description of the main findings of our study. Neurovascular coupling of the PCA was assessed using transcranial Doppler ultrasound, measuring changes in PCA BFV in response to a visual stimulus paradigm. The NVU, composed of neurons, astrocytes and vascular smooth muscle cells, is depicted at the capillary level. In this MS cohort, patients with lower disability (EDSS <2) and better cognitive performance exhibited a significant inverse association between NVC and WMH burden, such that preserved NVC was associated with lower WMH. Abbreviations: BFV= blood flow velocity; EDSS= Expanded Disability Status Scale; MS= multiple sclerosis; NVC= neurovascular coupling; NVU= neurovascular unit; PCA= posterior cerebral artery; WMH= white matter hyperintensities.

This study builds on our previous findings of cerebral autoregulation (CA) in MS where we demonstrated that more efficient CA was associated with higher GM volume, lower WMH burden, and better cognitive performance in patients with MS, independent of vascular risk factors and disease duration.^37^ We now extend that work and show that there is also a significant inverse relationship between NVC in the PCA and WMH burden in MS patients with lower disability (EDSS<2), with NVC PCA inversely associated with WMH in patients with higher executive and global cognitive scores (Figure 4). Collectively, these studies support the contribution of small vessel function to clinical and radiographic manifestations of MS.

While previous studies show that NVC is impaired in neurological disorders, including stroke^38^ and Alzheimer’s disease,^39^ as well as in healthy aging,^40^ data on NVC are limited and conflicting in MS. Two studies used TCD to measure NVC in MS patients during disease exacerbation.^41,42^ Both studies showed higher NVC and lower resting CBF compared with controls. In a follow-up study, NVC was reduced after repeated relapses, suggesting NVU damage in progressive disease.^43^ Other studies examined the effect of methylprednisolone and natalizumab infusion on NVC. While methylprednisolone was associated with reduced CBF^44^ and natalizumab showed elevated initial NVC response,^45^ neither treatment significantly altered overall NVC. Reduced CBF after methylprednisolone may be attributed to an anti-inflammatory effect decreasing vasoactive amines.^44^ Enhanced NVC after initial natalizumab infusion might be explained by reducing leukocytes, hence limiting their interference with endothelial function and NO bioavailability.^45^

Functional MRI (fMRI) studies investigating NVC in MS have also reported inconsistent results. NVC can be assessed using arterial spin labelling (ASL), conventional fMRI blood oxygen level-dependent (BOLD) imaging, or calibrated BOLD combined with ASL, to demonstrate CBF changes in response to neuronal activation.^46^ Two studies report significantly attenuated peak BOLD signal amplitudes in motor and visual cortices of RR MS patients compared to controls,^47,48^ while a study of 38 RR MS patients (mean EDSS score of 3) found no significant CBF differences during visual stimulation.^49^ Absence of control groups, different methodologies, and patient heterogeneity have significantly limited conclusions about NVC in MS based on existing studies.

In our MS cohort, a predominantly relapsing-remitting sample, impaired NVC in the PCA was associated with WMH burden, but only in patients with lower disability levels (EDSS <2). We propose that PCA NVC efficiency may serve as a clinically informative biomarker, capable of identifying MS patients who are more resilient to WM injury in early disease stages.

Brain functional plasticity plays an important role in MS progression. Resting-state fMRI studies have shown post-inflammatory reorganization via cortical recruitment, limiting functional impairment when structural damage is low.^50^ However, as disease progresses, this adaptive mechanism may become ineffective as cortical plasticity is exhausted,^51^ with progressive reduction in resting state connectivity from early to advanced disease associated with WM tract disruption^52^ and increased WMH burden.^53^ Additionally, cumulative NVU loss with disease progression,^43^ further impairs the coordination between neural activity and CBF, contributing to metabolic imbalance, oligodendrocyte death and further WM damage.^18^ These observations collectively suggest that the progressive loss of neurovascular function with MS disease progression may distinguish patients with differential vulnerability to structural injury.

Our data show that in MS patients with higher disability (EDSS≥2), NVC is not associated with WMH burden, supporting the notion that this biomarker may be stage-dependent. Disability accumulation in RR MS is attributed to an underlying progressive disease course,^2^ with pre-existing disability and older age constituting principal risk factors for further deterioration.^54^ Disability improvement appears restricted to initial MS years and following treatment initiation.^55^ Moreover, disease-modifying treatments have shown greater benefit in patients with lower disability.^56^ As MS progresses, determinants of WM damage shift. While inflammatory response declines, functional mechanisms like NVC may deteriorate due to cumulative damage and age-related changes.^57^

Previous studies of NVC and WM injury in patients with MS have not explicitly stratified analysis by disability level. An fMRI study of 32 MS patients (mean EDSS 3) found that reduced NVC variation in primary motor cortex during finger-tapping correlated with greater WM damage, measured using DTI.^58^ This study revealed a disability-dependent association, where NVC was impaired in MS participants with motor deficits. Our findings extend these observations by demonstrating that the relationship between NVC efficiency and conventional WM lesion burden and volume varies across disability strata, supporting the potential utility of NVC measures for risk stratification.

We also observed that in MS patients with higher global cognition and executive function, enhanced NVC was associated with lower WMH burden. This relationship has been documented in CSVD patients with preserved cognitive function.^59,60^ In MS, preserved NVC has been linked to attenuated cognitive slowing and reduced WM damage.^48,49^ Conversely, neurovascular uncoupling may exacerbate WMH and cognitive decline, by limiting adequate perfusion and metabolic support to neural networks.^49^ fMRI studies reveal that MS patients with higher cognitive function often show more efficient cortical network recruitment, potentially reflecting adaptive neurovascular responses that mitigate structural damage.^51^ Taken together these findings suggest that better cognition and less disability may define a subgroup of MS patients in whom improved NVC is associated with lower WM damage, a potentially resilient phenotype worth prospectively characterizing.

We also show that better NVC was associated with lower WM volume in patients with higher global cognitive score. Some patients with WM volume loss might maintain higher cognitive scores due to more efficient NVC in critical brain regions.^61^ Alternatively, this might represent WM pseudoatrophy, which can occur in MS after initiation of effective anti-inflammatory disease-modifying therapy.^62^ Further studies are needed to clarify these relationships.

Our study did not reveal any association between NVC and GM, regardless of disability status or cognitive performance. GM and WM differ in microvascular architecture, with GM exhibiting higher vascular density.^63^ NVC also varies regionally, due to differences in cellular composition and NVU distribution.^64^ WM appears particularly vulnerable: its endothelial cells lack CD44, increasing blood-brain barrier (BBB) disruption during neuroinflammation.^65^ Brain watershed regions, with lower perfusion and oxygen tension, are more prone to accumulate demyelinated plaques and have less effective repair mechanisms.^66^ Age-related vascular lesions also preferentially distribute in these areas.^67^ This convergence of NVC vulnerability and tissue susceptibility in WM may explain the distinct associations observed. Further longitudinal studies are needed to clarify this relationship.

Our reliance on conventional structural MRI represents an important limitation. WMH volume and volumetric measures capture macroscopic lesion burden but cannot identify microstructural change within normal-appearing WM, which may already be substantial in early MS.^68^ Our findings should therefore be interpreted as reflecting conventional lesion burden rather than the full spectrum of WM microstructural changes. Future work should extend these analyses using diffusion MRI metrics (fractional anisotropy, mean diffusivity, axial and radial diffusivity), which are sensitive to microstructural injury and have shown discriminatory value even in MS patients with low disability.^69^ Such techniques must, however, account for the methodological vulnerabilities of diffusion MRI: beyond random noise, diffusion metrics are affected by spatially dependent systematic bias arising from magnetic field gradient nonuniformity, which can distort diffusion estimates, tractography, and derived structure-function relationships.^70^ This is particularly important when interpreting subtle neurovascular or compensatory mechanisms in early MS, where effect sizes are small and may be confounded by these acquisition-dependent systematic effects.^70^ Correction frameworks addressing these effects, including the generalized Stejskal-Tanner formulation for non-uniform gradients^71^ and B-matrix spatial distribution calibration^69^ are relevant for future neurovascular microstructural studies in MS.

While our results provide valuable insights into NVC impairment in MS, underlying mechanisms remain elusive. Studies suggest several possible causes of NVC damage in MS, including dysfunctional mitochondria,^72^ oxidative injury,^73^ virtual hypoxia,^74^ astroglial toxicity,^75^ BBB permeability increase,^76^ and local inflammation across vessels.^77^

Our study did not find an association between NVC MCA and WMH burden, suggesting this relationship may be specific to PCA territory. Two potential mechanisms may explain this: varying capacity of task-induced CBF increase and inherent susceptibility of posterior cerebral regions. In healthy subjects, CBF increase after stimulation results in more pronounced PCA BFV increase (10-20%) compared to MCA BFV increase (5-8%).^13^ The visual stimulation task used for the PCA is straightforward, potentially eliciting a stronger CBF response compared to N-back used for MCA assessment. Moreover, the PCA supplies posterior brain regions with high metabolic activity.^78^ These factors suggest that NVC assessment in the PCA might yield a more robust and sensitive response.

This study has significant limitations. First, the cross-sectional design at a single center precludes establishing causal relationships and our data should be considered exploratory. Lack of a control group limits direct comparisons and constrains interpretations. Relying on clinical MRI potentially underestimated structural changes, given the possible one-year interval between TCD and MRI evaluations (median 52 days). While exclusion of patients with recent relapses or steroid use likely mitigated this, undetected lesion progression cannot be eliminated. Future studies should incorporate healthy controls, ensure concurrent MRI imaging and TCD assessments, and use longitudinal designs.

## 5. Conclusions

In conclusion, we show PCA NVC as a potential biomarker of conventional WM lesion burden in MS, with particular discriminatory value in less disabled patients and those with better cognitive performance. As these findings derive from a cross-sectional study of relatively small, single-center cohort, they should be regarded as preliminary and require confirmation in larger prospective longitudinal studies. NVC efficiency may help identify a clinically resilient MS subgroup and support risk stratification in future longitudinal and interventional studies, serving as a low-cost, non-invasive complement to conventional neuroimaging. Future studies to elucidate the mechanisms underlying the observed association between NVC and WM damage in patients with MS may lead to novel therapeutic targets.

## Supplemental material

Supplemental material - Preserved neurovascular coupling in early multiple sclerosis is associated with reduced white matter lesion burden Supplemental material for Preserved neurovascular coupling in early multiple sclerosis is associated with reduced white matter lesion burden by Cristina Duque, João Sargento-Freitas, Roumen Balabanov, Bruce Cohen, and Farzaneh A. Sorond in Therapeutic Advances in Neurological Disorders.

## Data Availability

Further anonymized data can be made available upon reasonable request from a qualified investigator to the corresponding author.*

## References

[1] Noseworthy JH Lucchinetti C Rodriguez M , et al. Multiple sclerosis. N Engl J Med 2000; 343: 938–952. 10.1056/NEJM200009283431307 11006371

[2] Kappos L Wolinsky JS Giovannoni G , et al. Contribution of Relapse-Independent Progression vs Relapse-Associated Worsening to Overall Confirmed Disability Accumulation in Typical Relapsing Multiple Sclerosis in a Pooled Analysis of 2 Randomized Clinical Trials. JAMA Neurol 2020; 77: 1132–1140. 10.1001/jamaneurol.2020.1568 32511687 PMC7281382

[3] Lublin FD Reingold SC . Defining the clinical course of multiple sclerosis: results of an international survey. National Multiple Sclerosis Society (USA) Advisory Committee on Clinical Trials of New Agents in Multiple Sclerosis. Neurology 1996; 46: 907–911. 10.1212/wnl.46.4.907 8780061

[4] Hawker K . Progressive multiple sclerosis: characteristics and management. Neurol Clin 2011; 29: 423–434. 10.1016/j.ncl.2011.01.002 21439451

[5] Frohman EM Racke MK Raine CS . Multiple sclerosis--the plaque and its pathogenesis. N Engl J Med 2006; 354: 942–955. 10.1056/NEJMra052130 16510748

[6] Kuhlmann T Moccia M Coetzee T , et al. Multiple sclerosis progression: time for a new mechanism-driven framework. Lancet Neurol 2023; 22: 78–88. 10.1016/S1474-4422(22)00289-7 36410373 PMC10463558

[7] Geraldes R Esiri MM Perera R , et al. Vascular disease and multiple sclerosis: a post-mortem study exploring their relationships. Brain 2020; 143: 2998–3012. 10.1093/brain/awaa255 32875311

[8] D’haeseleer M Hostenbach S Peeters I , et al. Cerebral hypoperfusion: a new pathophysiologic concept in multiple sclerosis? J Cereb Blood Flow Metab 2015; 35: 1406–1410. 10.1038/jcbfm.2015.131 26104292 PMC4640326

[9] D’haeseleer M Cambron M Vanopdenbosch L , et al. Vascular aspects of multiple sclerosis. The Lancet Neurology 2011; 10: 657–666. 10.1016/S1474-4422(11)70105-3 21683931

[10] Ge Y Law M Johnson G , et al. Dynamic susceptibility contrast perfusion MR imaging of multiple sclerosis lesions: characterizing hemodynamic impairment and inflammatory activity. AJNR Am J Neuroradiol 2005; 26: 1539–1547. 15956527 PMC8149080

[11] De Keyser J Steen C Mostert JP , et al. Hypoperfusion of the Cerebral White Matter in Multiple Sclerosis: Possible Mechanisms and Pathophysiological Significance. J Cereb Blood Flow Metab 2008; 28: 1645–1651. 10.1038/jcbfm.2008.72 18594554

[12] Mahad DJ Ziabreva I Campbell G , et al. Mitochondrial changes within axons in multiple sclerosis. Brain 2009; 132: 1161–1174. 10.1093/brain/awp046 19293237 PMC3605917

[13] Phillips AA Chan FH Zheng MMZ , et al. Neurovascular coupling in humans: Physiology, methodological advances and clinical implications. J Cereb Blood Flow Metab 2016; 36: 647–664. 10.1177/0271678X15617954 26661243 PMC4821024

[14] Zlokovic BV . Neurovascular pathways to neurodegeneration in Alzheimer’s disease and other disorders. Nat Rev Neurosci 2011; 12: 723–738. 10.1038/nrn3114 22048062 PMC4036520

[15] Plumb J McQuaid S Mirakhur M , et al. Abnormal endothelial tight junctions in active lesions and normal-appearing white matter in multiple sclerosis. Brain Pathol 2002; 12: 154–169. 10.1111/j.1750-3639.2002.tb00430.x 11958369 PMC8095734

[16] Kirk J Plumb J Mirakhur M , et al. Tight junctional abnormality in multiple sclerosis white matter affects all calibres of vessel and is associated with blood-brain barrier leakage and active demyelination. J Pathol 2003; 201: 319–327. 10.1002/path.1434 14517850

[17] Geraldes R Esiri MM DeLuca GC , et al. Age-related small vessel disease: a potential contributor to neurodegeneration in multiple sclerosis. Brain Pathol 2017; 27: 707–722. 10.1111/bpa.12460 27864848 PMC8029132

[18] Cashion JM Young KM Sutherland BA . How does neurovascular unit dysfunction contribute to multiple sclerosis? Neurobiol Dis 2023; 178: 106028. 10.1016/j.nbd.2023.106028 36736923

[19] Aaslid R Markwalder TM Nornes H . Noninvasive transcranial Doppler ultrasound recording of flow velocity in basal cerebral arteries. J Neurosurg 1982; 57: 769–774. 10.3171/jns.1982.57.6.0769 7143059

[20] Aaslid R . Visually evoked dynamic blood flow response of the human cerebral circulation. Stroke 1987; 18: 771–775. 10.1161/01.str.18.4.771 3299883

[21] Paulson OB Hasselbalch SG Rostrup E , et al. Cerebral blood flow response to functional activation. J Cereb Blood Flow Metab 2010; 30: 2–14. 10.1038/jcbfm.2009.188 19738630 PMC2872188

[22] Thompson AJ Banwell BL Barkhof F , et al. Diagnosis of multiple sclerosis: 2017 revisions of the McDonald criteria. Lancet Neurol 2018; 17: 162–173. 10.1016/S1474-4422(17)30470-2 29275977

[23] Montalban X Lebrun-Frénay C Oh J , et al. Diagnosis of multiple sclerosis: 2024 revisions of the McDonald criteria. Lancet Neurol 2025; 24: 850–865. 10.1016/S1474-4422(25)00270-4 40975101

[24] Kurtzke JF . Rating neurologic impairment in multiple sclerosis: an expanded disability status scale (EDSS). Neurology 1983; 33: 1444–1452. 10.1212/wnl.33.11.1444 6685237

[25] Sorond FA Hurwitz S Salat DH , et al. Neurovascular coupling, cerebral white matter integrity, and response to cocoa in older people. Neurology 2013; 81: 904–909. 10.1212/WNL.0b013e3182a351aa 23925758 PMC3885215

[26] Sorond FA Kiely DK Galica A , et al. Neurovascular coupling is impaired in slow walkers: The MOBILIZE Boston Study. Annals of Neurology 2011; 70: 213–220. 10.1002/ana.22433 21674588 PMC3152682

[27] Monteiro A Castro P Pereira G , et al. Cerebral blood flow regulation and cognitive performance in hypertension. J Cereb Blood Flow Metab 2024; 271678X241254680: 1277–1287. 10.1177/0271678X241254680 PMC11542125 38738526

[28] Rosengarten B Huwendiek O Kaps M . Neurovascular coupling in terms of a control system: validation of a second-order linear system model. Ultrasound Med Biol 2001; 27: 631–635. 10.1016/s0301-5629(01)00355-6 11397527

[29] Monteiro A Castro P Pereira G , et al. Neurovascular Coupling Is Impaired in Hypertensive and Diabetic Subjects Without Symptomatic Cerebrovascular Disease. Front Aging Neurosci 2021; 13: 728007. 10.3389/fnagi.2021.728007 34690741 PMC8526560

[30] Cutter GR Baier ML Rudick RA , et al. Development of a multiple sclerosis functional composite as a clinical trial outcome measure. Brain 1999; 122(Pt 5): 871–882. 10.1093/brain/122.5.871 10355672

[31] Lamers I Feys P . Assessing upper limb function in multiple sclerosis. Mult Scler 2014; 20: 775–784. 10.1177/1352458514525677 24664300

[32] Mahinrad S Shownkeen M Sedaghat S , et al. Vascular health across young adulthood and midlife cerebral autoregulation, gait, and cognition. Alzheimers Dement 2021; 17: 745–754. 33283978 10.1002/alz.12246 PMC11932044

[33] Jor’dan AJ Manor B Iloputaife I , et al. Diminished Locomotor Control Is Associated With Reduced Neurovascular Coupling in Older Adults. J Gerontol A Biol Sci Med Sci 2020; 75: 1516–1522. 10.1093/gerona/glz006 30629129 PMC7357586

[34] Manjón JV Coupé P Martí-Bonmatí L , et al. Adaptive non-local means denoising of MR images with spatially varying noise levels. J Magn Reson Imaging 2010; 31: 192–203. 10.1002/jmri.22003 20027588

[35] Tustison NJ Avants BB Cook PA , et al. N4ITK: improved N3 bias correction. IEEE Trans Med Imaging 2010; 29: 1310–1320. 10.1109/TMI.2010.2046908 20378467 PMC3071855

[36] Schmidt P . Bayesian inference for structured additive regression models for large-scale problems with applications to medical imaging. Ludwig-Maximilians-Universität München, 2017. 10.5282/EDOC.20373. Epub ahead of print.

[37] Duque C Mahinrad S Sedaghat S , et al. Cerebrovascular hemodynamics association with brain structure and function in Multiple Sclerosis. Mult Scler Relat Disord 2024; 91: 105882. 10.1016/j.msard.2024.105882 39276598 PMC11835024

[38] Salinet ASM Haunton VJ Panerai RB , et al. A systematic review of cerebral hemodynamic responses to neural activation following stroke. J Neurol 2013; 260: 2715–2721. 10.1007/s00415-013-6836-z 23315262

[39] Nicolakakis N Hamel E . Neurovascular function in Alzheimer’s disease patients and experimental models. J Cereb Blood Flow Metab 2011; 31: 1354–1370. 10.1038/jcbfm.2011.43 21468088 PMC3130325

[40] Flück D Beaudin AE Steinback CD , et al. Effects of aging on the association between cerebrovascular responses to visual stimulation, hypercapnia and arterial stiffness. Front Physiol 2014; 5: 49. 10.3389/fphys.2014.00049 24600398 PMC3928624

[41] Uzuner N Ozkan S Gücüyener D , et al. Cerebral blood flow velocity changes to visual stimuli in patients with multiple sclerosis. Mult Scler 2002; 8: 217–221. 10.1191/1352458502ms798oa 12120693

[42] Tekgöl UG Uzuner N . Neurovascular coupling in patients with relapsing-remitting multiple sclerosis. Clinical Neurology and Neurosurgery 2016; 146: 24–28. 10.1016/j.clineuro.2016.04.020 27136094

[43] Nevzat U Gulnur TU . Neurovascular Reactivity after Repeated Attacks in Patients with Multiple Sclerosis. International Journal of Multiple Sclerosis and Related Disorders 2017; 1: 1–5.

[44] Ozkan S Uzuner N Kutlu C , et al. The effect of methylprednisolone treatment on cerebral reactivity in patients with multiple sclerosis. J Clin Neurosci 2006; 13: 214–217. 10.1016/j.jocn.2005.03.023 16503485

[45] Reinhard M Rosengarten B Kirchhoff L , et al. Natalizumab and regulation of cerebral blood flow: results from an observational study. Eur Neurol 2010; 64: 124–128. 10.1159/000316765 20664205

[46] Vestergaard MB Frederiksen JL Larsson HBW , et al. Cerebrovascular Reactivity and Neurovascular Coupling in Multiple Sclerosis-A Systematic Review. Front Neurol 2022; 13: 912828. 10.3389/fneur.2022.912828 35720104 PMC9198441

[47] Hubbard NA Turner M Hutchison JL , et al. Multiple sclerosis-related white matter microstructural change alters the BOLD hemodynamic response. J Cereb Blood Flow Metab 2016; 36: 1872–1884. 10.1177/0271678X15615133 26661225 PMC5094308

[48] Turner MP Hubbard NA Sivakolundu DK , et al. Preserved canonicality of the BOLD hemodynamic response reflects healthy cognition: Insights into the healthy brain through the window of Multiple Sclerosis. Neuroimage 2019; 190: 46–55. 10.1016/j.neuroimage.2017.12.081 29454932 PMC6093806

[49] Sivakolundu DK West KL Zuppichini M , et al. The neurovascular basis of processing speed differences in humans: A model-systems approach using multiple sclerosis. Neuroimage 2020; 215: 116812. 10.1016/j.neuroimage.2020.116812 32276075

[50] Rocca MA Colombo B Falini A , et al. Cortical adaptation in patients with MS: a cross-sectional functional MRI study of disease phenotypes. Lancet Neurol 2005; 4: 618–626. 10.1016/S1474-4422(05)70171-X 16168930

[51] Rocca MA Amato MP De Stefano N , et al. Clinical and imaging assessment of cognitive dysfunction in multiple sclerosis. Lancet Neurol 2015; 14: 302–317. 10.1016/S1474-4422(14)70250-9 25662900

[52] Rocca MA Valsasina P Absinta M , et al. Default-mode network dysfunction and cognitive impairment in progressive MS. Neurology 2010; 74: 1252–1259. 10.1212/WNL.0b013e3181d9ed91 20404306

[53] Rocca MA Valsasina P Leavitt VM , et al. Functional network connectivity abnormalities in multiple sclerosis: Correlations with disability and cognitive impairment. Mult Scler 2018; 24: 459–471. 10.1177/1352458517699875 28294693

[54] Lublin FD Häring DA Ganjgahi H , et al. How patients with multiple sclerosis acquire disability. Brain 2022; 145: 3147–3161. 10.1093/brain/awac016 35104840 PMC9536294

[55] Lassmann H van Horssen J Mahad D . Progressive multiple sclerosis: pathology and pathogenesis. Nat Rev Neurol 2012; 8: 647–656. 10.1038/nrneurol.2012.168 23007702

[56] Diouf I Malpas CB Sharmin S , et al. Variability of the response to immunotherapy among subgroups of patients with multiple sclerosis. Eur J Neurol 2023; 30: 1014–1024. 10.1111/ene.15706 36692895 PMC10946605

[57] Mahad DH Trapp BD Lassmann H . Pathological mechanisms in progressive multiple sclerosis. Lancet Neurol 2015; 14: 183–193. 10.1016/S1474-4422(14)70256-X 25772897

[58] West KL Sivakolundu DK Zuppichini MD , et al. Altered task-induced cerebral blood flow and oxygen metabolism underlies motor impairment in multiple sclerosis. J Cereb Blood Flow Metab 2021; 41: 182–193. 32126873 10.1177/0271678X20908356 PMC7747162

[59] Ruan Z Sun D Zhou X , et al. Altered neurovascular coupling in patients with vascular cognitive impairment: a combined ASL-fMRI analysis. Front Aging Neurosci 2023; 15: 1224525. 10.3389/fnagi.2023.1224525 37416325 PMC10320594

[60] Li H Chai C Xie Y , et al. Disturbed neurovascular coupling in patients with white matter hyperintensities: potential biomarker for cognitive impairment. Neuroradiology 2024; 66: 1967–1978. 10.1007/s00234-024-03459-z 39616265

[61] Lageman SB Jolly A Sahi N , et al. Explaining cognitive function in multiple sclerosis through networks of grey and white matter features: a joint independent component analysis. J Neurol 2025; 272: 142. 10.1007/s00415-024-12795-2 39812878 PMC11735591

[62] Vidal-Jordana A Sastre-Garriga J Pérez-Miralles F , et al. Early brain pseudoatrophy while on natalizumab therapy is due to white matter volume changes. Mult Scler 2013; 19: 1175–1181. 10.1177/1352458512473190 23319072

[63] Cavaglia M Dombrowski SM Drazba J , et al. Regional variation in brain capillary density and vascular response to ischemia. Brain Res 2001; 910: 81–93. 10.1016/s0006-8993(01)02637-3 11489257

[64] Bernier L-P Brunner C Cottarelli A , et al. Location Matters: Navigating Regional Heterogeneity of the Neurovascular Unit. Front Cell Neurosci 2021; 15: 696540. 10.3389/fncel.2021.696540 34276312 PMC8277940

[65] Flynn KM Michaud M Madri JA . CD44 deficiency contributes to enhanced experimental autoimmune encephalomyelitis: a role in immune cells and vascular cells of the blood-brain barrier. Am J Pathol 2013; 182: 1322–1336. 10.1016/j.ajpath.2013.01.003 23416161 PMC3620422

[66] Holland CM Charil A Csapo I , et al. The relationship between normal cerebral perfusion patterns and white matter lesion distribution in 1,249 patients with multiple sclerosis. J Neuroimaging 2012; 22: 129–136. 10.1111/j.1552-6569.2011.00585.x 21447022

[67] Haider L Zrzavy T Hametner S , et al. The topograpy of demyelination and neurodegeneration in the multiple sclerosis brain. Brain 2016; 139: 807–815. 10.1093/brain/awv398 26912645 PMC4766379

[68] Griffin CM Chard DT Ciccarelli O , et al. Diffusion tensor imaging in early relapsing-remitting multiple sclerosis. Mult Scler 2001; 7: 290–297. 10.1177/135245850100700504 11724444

[69] Krzyzak AT Lasek J Slowik A . Diagnostic performance of a multi-shell DTI protocol and its subsets with B-matrix spatial distribution correction in differentiating early multiple sclerosis patients from healthy controls. Front Neurol 2025; 16: 1618582. 10.3389/fneur.2025.1618582 40800684 PMC12340240

[70] Mesri HY David S Viergever MA , et al. The adverse effect of gradient nonlinearities on diffusion MRI: From voxels to group studies. Neuroimage 2020; 205: 116127. 10.1016/j.neuroimage.2019.116127 31476431

[71] Borkowski K Krzyżak AT . The generalized Stejskal-Tanner equation for non-uniform magnetic field gradients. J Magn Reson 2018; 296: 23–28. 10.1016/j.jmr.2018.08.010 30195715

[72] Patergnani S Fossati V Bonora M , et al. Mitochondria in Multiple Sclerosis: Molecular Mechanisms of Pathogenesis. Int Rev Cell Mol Biol 2017; 328: 49–103. 10.1016/bs.ircmb.2016.08.003 28069137

[73] Fischer MT Sharma R Lim JL , et al. NADPH oxidase expression in active multiple sclerosis lesions in relation to oxidative tissue damage and mitochondrial injury. Brain 2012; 135: 886–899. 10.1093/brain/aws012 22366799 PMC3286337

[74] Trapp BD Stys PK . Virtual hypoxia and chronic necrosis of demyelinated axons in multiple sclerosis. Lancet Neurol 2009; 8: 280–291. 10.1016/S1474-4422(09)70043-2 19233038

[75] Liddelow SA Guttenplan KA Clarke LE , et al. Neurotoxic reactive astrocytes are induced by activated microglia. Nature 2017; 541: 481–487. 10.1038/nature21029 28099414 PMC5404890

[76] Cramer SP Simonsen H Frederiksen JL , et al. Abnormal blood-brain barrier permeability in normal appearing white matter in multiple sclerosis investigated by MRI. Neuroimage Clin 2014; 4: 182–189. 10.1016/j.nicl.2013.12.001 24371801 PMC3872721

[77] Angleys H Østergaard L Jespersen SN . The effects of capillary transit time heterogeneity (CTH) on brain oxygenation. J Cereb Blood Flow Metab 2015; 35: 806–817. 10.1038/jcbfm.2014.254 25669911 PMC4420854

[78] Volpi T Lee JJ Vlassenko AG , et al. The brain’s ‘dark energy’ puzzle upgraded: [18F]FDG uptake, delivery and phosphorylation, and their coupling with resting-state brain activity. J Cereb Blood Flow Metab 2025; 45: 1799–1815. 10.1177/0271678x251329707 40370305 PMC12081390

