# Supplementary material for: Preserved neurovascular coupling in early multiple sclerosis is associated with reduced white matter lesion burden
Source: Ther Adv Neurol Disord. 2026 Aug 24;19:17562864261481753. doi: 10.1177/17562864261481753 (PMC13504116; doi:10.1177/17562864261481753)
Supplement: Supplementary file 1 [file sj-pdf-1-tan-10.1177_17562864261481753.pdf]

Table S1. Baseline characteristics of participants in the entire sample and divided across low versus high cognition domains: Attention, Executive, Memory and Global

	All	Attention		p	Executive		p	Memory		p	Global		p
	n=76	Low n=38	High n=38		Low n=38	High n=38		Low n=38	High n=38		Low n=38	High n=38	
**Demographics**													
Age, years[+]	44 (10)	46 (11)	43 (10)	0.224	47 (10)	42 (10)	0.033*	46 (11)	43 (10)	0.170	48 (9)	41 (10)	0.002*
Male, n (%)	21 (28)	8 (21)	13 (34)	0.200	10 (27)	11 (29)	0.853	11 (29)	10 (26)	0.798	10 (26)	11 (30)	0.742
White, n (%)	71 (93)	33 (87)	38 (100)	0.021*	34 (92)	37 (97)	0.291	34 (90)	37 (97)	0.165	34 (90)	37 (100)	0.043*
**Multiple Sclerosis Clinical Characteristics**													
RR form, n (%)	71 (93)	35 (92)	36 (95)	0.841	35 (95)	35 (92)	0.852	34 (90)	37 (97)	0.209	34 (90)	36 (97)	0.218
Disease duration[++]	8 (11)	10 (7)	3 (10)	0.013*	11 (14)	5 (9)	0.001*	9 (13)	6 (10)	0.855	11 (12)	5 (11)	0.009*
EDSS[++]	2.0 (4.0)	2.0 (4.0)	1.0 (2.0)	0.003*	2.0 (4.5)	1.5 (2.0)	0.004*	2.0 (4.0)	2.0 (1.0)	0.050	3.0 (4.0)	1.5 (2.0)	<0.001*
**Vascular Risk Factors**													
HTN, n (%)	8 (11)	8 (21)	0 (0)	0.003*	6 (16)	2 (5)	0.124	7 (18)	1 (3)	0.025*	7 (18)	1 (3)	0.027*
Diabetes, n (%)	8 (11)	8 (21)	0 (0)	0.003*	7 (19)	1 (3)	0.022*	7 (18)	1 (3)	0.025*	7 (18)	1 (3)	0.027*
Current Smoker, n (%)	6 (8)	4 (11)	2 (5)	0.395	3 (8)	3 (8)	0.973	2 (5)	4 (11)	0.395	2 (5)	4 (11)	0.376
Dyslipidemia, n (%)	9 (12)	9 (24)	0 (0)	0.001*	8 (22)	1 (3)	0.011*	7 (18)	2 (5)	0.076	8 (21)	1 (3)	0.014*
BMI ≥ 25kg/m^2, n (%)	43 (57)	26 (68)	17 (44)	0.037*	24 (65)	18 (47)	0.127	24 (63)	19 (50)	0.247	25 (66)	17 (46)	0.083
**Neurovascular Coupling**													
MCA MFV, cm/s, % [++]	3.3 (6.1)	1.4 (5.9)	4.3 (5.7)	0.049*	1.9 (6.6)	3.8 (5.7)	0.203	2.0 (7.1)	2.8 (6.3)	0.408	2.7 (6.3)	3.5 (5.9)	0.331
PCA MFV, cm/s, % [++]	5.4 (10.9)	4.8 (11.6)	4.8 (10.2)	0.644	6.3 (11.0)	4.8 (10.8)	0.474	2.8 (9.9)	7.9 (10.8)	0.342	5.6 (12.0)	5.4 (9.9)	0.951
**Manual Dexterity**													
9HPT, DH, s[++]	21 (7)	24 (6)	19 (3)	0.003*	24 (7)	20 (6)	0.005*	24 (7)	19 (5)	0.035*	24 (7)	19 (3)	<0.001*
9HPT, NDH, s[++]	22 (8)	24 (12)	21 (5)	0.025*	24 (13)	22 (4)	0.005*	24 (11)	21 (5)	0.004*	25 (12)	21 (4)	<0.001*

**Cognitive Parameters**												
Education, years++	16 (4)	16 (5)	16 (2)	0.102	16 (3)	16 (2)	0.050*	16 (3)	16 (3)	0.151	16 (3)	16 (2)	0.008*
**Gait Velocity**												
Self-pace, cm/s++	121 (47)	101 (50)	131 (29)	0.009*	110 (57)	131 (26)	0.048*	107 (47)	126 (33)	0.051	107 (53)	134 (26)	<0.001*
Fast-pace, cm/s++	161 (56)	149 (63)	181 (39)	0.014*	153 (57)	175 (57)	0.006*	151 (64)	177 (35)	0.022*	145 (51)	182 (48)	<0.001*
Self-pace DT, cm/s++	79 (53)	64 (39)	95 (34)	0.016*	62 (37)	95 (41)	0.002*	71 (34)	91 (57)	0.093	67 (41)	94 (44)	0.001*
Fast -pace DT, cm/s++	143 (59)	122 (57)	157 (27)	0.003*	118 (54)	153 (37)	0.001*	131 (59)	150 (24)	0.008*	107 (52)	153 (32)	<0.001*
**MRI Measures**												
White matter volume+	0.30 (0.02)	0.29 (0.03)	0.30 (0.02)	0.010*	0.28 (0.02)	0.30 (0.02)	0.001*	0.29 (0.02)	0.30 (0.02)	0.049	0.29 (0.03)	0.30 (0.02)	0.003*
WMH ++	0.003 (0.005)	0.006 (0.01)	0.002 (0.003)	0.024*	0.005 (0.01)	0.002 (0.003)	0.010*	0.004 (0.009)	0.002 (0.003)	0.030*	0.006 (0.012)	0.002 (0.002)	<0.001*
Gray matter volume+	0.41 (0.03)	0.41 (0.03)	0.42 (0.03)	0.058	0.40 (0.03)	0.42 (0.03)	0.019*	0.40 (0.02)	0.42 (0.04)	0.013*	0.40 (0.03)	0.43 (0.03)	0.002*

+Values are presented as mean (standard deviation).
++Values are presented as median (interquartile range).
*p-value <0.05.

P values were calculated using t test or Mann-Whitney U test for continuous variables, and χ^2 test for categorical variables.
Dyslipidemia defined by a total cholesterol ≥ 200 mg/dL.
MRI measures normalized for intracranial volume.

Abbreviations: BMI= Body Mass Index; DH= dominant hand; DT= dual task; EDSS= Expanded Disability Status Scale; GM= gray matter; HTN= hypertension; MCA= middle cerebral artery; MFV= mean blood flow velocity; MRI= magnetic resonance imaging; NDH= non-dominant hand; NVC= Neurovascular Coupling; PCA= posterior cerebral artery; RR= relapsing-remitting; s= seconds; WMH= white matter hyperintensities; 9HPT= Nine-Hole Peg test.
